# Time till pleuropulmonary recurrence for mesothelioma and high grade appendiceal neoplasm after CRS/HIPEC

**DOI:** 10.1515/pp-2025-0010

**Published:** 2025-06-23

**Authors:** Tiffany Guadalupe Williams, Shoma Barat, David Lawson Morris

**Affiliations:** Department of Surgery, Peritonectomy Unit, St George Hospital, Sydney, Australia; Faculty of Medicine, Department of Surgery, University of New South Wales, St George Hospital, Sydney, Australia

**Keywords:** peritoneal malignancy, pleuropulmonary disease, recurrence

## Abstract

**Objectives:**

Cytoreductive surgery with heated intraperitoneal chemotherapy (CRS/HIPEC) requiring diaphragmatic stripping or resection may predispose to pleuropulmonary recurrence. This review was to assess the rates of pleuropulmonary recurrence after CRS/HIPEC for patients who had high-grade appendiceal neoplasm and meosthelioma.

**Methods:**

A retrospective review (September 1996–November 2021) at a single tertiary center identified 716 patients who underwent CRS/HIPEC with diaphragmatic intervention; 203 had high-grade appendiceal neoplasms and 63 had mesothelioma. Radiologic or pathologic evidence of pleuropulmonary recurrence was recorded. Time from CRS/HIPEC to chest recurrence was analyzed using Kaplan–Meier methods.

**Results:**

Twenty patients (12 appendiceal; 8 mesothelioma) developed pleuropulmonary recurrence. In the appendiceal cohort (mean age 51.5 years; median PCI 30), all 12 underwent bilateral diaphragm intervention (four with full-thickness resection) with CCR 0–1. Time to chest recurrence ranged from 0.3 to 82.8 months; half experienced early respiratory complications (e.g., pleural effusion, pneumothorax). In the mesothelioma cohort (mean age 44.9 years; median PCI 22.1), seven had bilateral stripping (two with resection) and one had unilateral stripping; CCR was 0–1. Recurrence occurred between 8.0 and 85.4 months (median ∼31.4 months); half had early respiratory compromise. No significant associations were observed between PCI, CCR, or extent of diaphragmatic intervention and recurrence risk, although ICU stay and CCR weakly correlated with recurrence in mesothelioma.

**Conclusions:**

Pleuropulmonary recurrence following CRS/HIPEC with diaphragm intervention is rare (2.7 %), with early recurrences suggesting occult thoracic involvement. Bilateral diaphragm manipulation was common among those with recurrence.

## Introduction

Cytoreductive surgery (CRS) and heated intraperitoneal chemotherapy (HIPEC) are the basis of treatment of peritoneal carcinomatosis, with evidence of increased overall survival and disease-free survival [1], [2], [3], [4]. Recurrence of disease is an unfortunate, but occasional occurrence in the post-operative setting. Common places of recurrence are areas of complex surgical intervention or occasionally incomplete cytoreduction, particularly over regions such as the diaphragm. Diaphragmatic intervention is growing in CRS as evidence demonstrates that while increased rates of post-operative morbidity, patients have greater overall survival compared to patients who have incomplete cytoreduction or palliative chemotherapy [].

Diaphragmatic intervention occurs in 50 % of CRS/HIPEC cases and consists of numerous different techniques [8]. Different diaphragmatic interventions include unilateral or bilateral intervention, diaphragm stripping, where the superficial disease is removed, or full thickness resection of the diaphragm, in which complete aspects of the diaphragm is removed, the thoracic cavity is entered, and repair or closure of the diaphragm is required [9].

The aims of this study were to investigate the time taken for pleural disease to prevail in patients who underwent CRS/HIPEC and had diaphragm involvement. The hypothesis of this investigation is that diaphragmatic intervention increases the risk of pleuropulmonary resection. A secondary assessment was of associated post-operative complications in patients who then developed pleural disease. The hypothesis of this investigation is that diaphragmatic intervention increases the risk of pleuropulmonary resection.

## Methodology

This was a retrospective cohort study conducted in a single high-volume centre, St George Hospital, Sydney from a prospectively maintained database. Ethics approval was obtained 2022/ETH02207: Clinical Studies in Abdominal and Peritoneal Cancers. The database was dated from September 1996 to November 2021 and included all patients who had undergone CRS/HIPEC for peritoneal based malignancies. Suitability for CRS/HIPEC is assessed based on disease histology, extent and ability to achieve complete cytoreduction, performance status and comorbidities. Subsequent CRS/HIPEC was performed utilising the principles established by Sugarbaker [10].

### Patient selection

All patients in this analysis were originally assessed pre-operatively for fitness for surgery with workup including basic blood tests, dihydropyrimidine dehydrogenase (DPYD) levels, tumour markers and computed topography imaging of chest, abdomen and pelvis. Magnetic resonance imaging of the liver and positron emission tomography were used depending on the patient’s disease characteristics and aetiology. All patients are discussed at a multidisciplinary meeting.

### Intra-operative care

Intraoperatively, all patients undergo cytoreductive surgery in accordance with the principals established by Sugarbaker [10]. The peritoneal cancer index (PCI) is calculated at the start of the operation to grade the volume of disease and completeness of cytoreduction (CC) recorded at the end of cytoreduction for macroscopic tumour left. Post CRS, HIPEC is administered using the coliseum (open) technique. The abdomen was initially primed with Dianeal PD4 (1.5 %) peritoneal dialysis solution, in later years switching to plasmalyte 148, and heated to 41.5 degree Celsius, with chemotherapy infused when reaching this temperature. Agents used includes mitomycin C (12.5 mg/m^2^) for 90 min, oxaliplatin (350 mg/m^2^) for 30 min or cisplatin (100 mg/m^2^) for 90 min, as prescribed by medical oncology depending on the specific aetiology.

### Data collection and statistical analysis

Demographics such as age, sex and American Society of Anaesthetists (ASA) score were recorded. Operative information such as PCI, CC score and HIPEC agent were collected. Length of stay in hospital and ICU were measured in days. Morbidity was defined using the Clavien–Dindo classification from Grades I to V, with grade III and IV complications defined as a major morbidity and grade V defined as a mortality.

All statistics were performed using IBM® SPSS® software Version 24. The level of significance (p) was set to 0.05 with p values less than 0.05 considered as statistically significant. Pearson’s correlation coefficient was calculated to assess association for preoperative and operative information.

The extent of peritoneal disease was documented via the peritoneal carcinomatosis index (PCI) intraoperatively, as described by Jacquet and Sugarbaker [10], 11]. Completeness of cytoreduction (CC) was recorded in a similar manner. Postoperative complications were graded according to the Claven–Dindo classification [12]. Prior surgical score was documented as described by Jacquet et al. [11]. There have been several modifications of nomenclature and pathological analysis of pseudomyxoma. We adhere to the most recent classification by Carr and colleagues [13].

### Surgical technique

In this cohort study, diaphragm interventions included patients who underwent diaphragm stripping or muscle resection. Our unit routinely performed complete diaphragmatic peritonectomy, and very rarely performed localised peritonectomy. However, if muscle involvement is present, local resection and repair is either performed primarily with mesh or using bovine pericardium. Intercostal catheters are routinely inserted in all patients undergoing diaphragmatic interventions for control of pleural effusions and pneumothoraces as a result of intervention.

## Analysis

Patients included in this study were selected dependent on whether they had radiological evidence of pleuropulmonary recurrence. Demographic data as outlined above was noted.

## Results

In a cohort of 716 number of patients who had diaphragm intervention, 203 patients’ histological subtype was high grade appendiceal neoplasm, and 63 patients had mesothelioma. A total of 20 patients had evidence of chest or pleural metastasis who had undergone diaphragm intervention, 12 of the high grade appendiceal neoplasms and eight of the mesothelioma patients.

### High grade appendiceal neoplasm

**Diagram 1:**
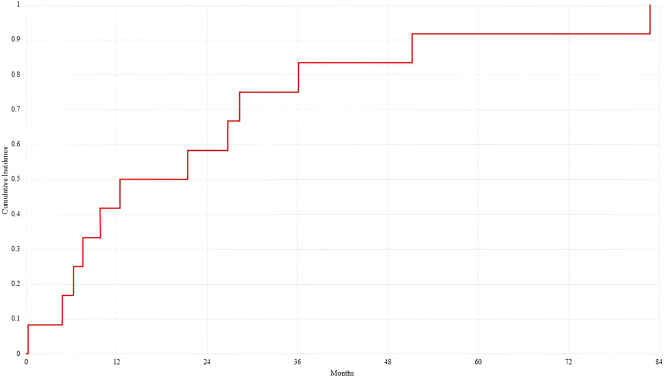
Kaplan–Meier curve demonstrating time till chest disease recurrence for patients with high grade appendiceal neoplasms.

Twelve patient’s tumour histopathological subtype was appendiceal adenocarcinoma (Table 1a). Six patients (50 %) were male. Age of the patients at time of operation ranged from 32.1 to 68.9, with a mean age of 51.5 years. Of all patients who had disease recurrence in the chest, all had bilateral intervention, with four patients requiring resection of the diaphragm. PCI score ranges from 16–36, with a median PCI of 30. All patients had either a CC score of 0 or 1. Time till chest recurrence, in months, ranged from 0.263 to 82.8 months (Diagram 1).

For pre-operative and operative characteristics, there was no statistical significance between pleuropulmonary recurrence and operative hours, days in intensive care unit (ICU), total hospital length of stay, PCI, or CC-score. There was weak correlation associated with gender (r^2^=−0.48, p<0.05) and age (r^2^=0.34, p<0.05), however limited clinical significance.

**Table 1a:** Operative characteristics of high grade appendiceal neoplasm patients who had recurrence in the form of chest disease.

Patient	Age (years)	Gender	PCI	CC score	Diaphragmatic intervention	Time till chest recurrence (months)
1	32.1	Female	23	0	Bilateral strip	0.263
2	34.14	Female	35	0	Bilateral strip, right sided resection	4.8
3	68.9	Female	35	1	Bilateral strip, bilateral repair	6.28
4	68.1	Male	36	0	Bilateral strip	7.52
5	53	Female	27	1	Bilateral strip, right sided resection	9.86
6	32	Male	35	0	Bilateral strip	12.42
7	59	Female	31	1	Bilateral strip	21.45
8	58.3	Male	36	1	Bilateral strip	26.74
9	41.2	Male	21	0	Right sided strip, left sided resection	28.3
10	46	Female	37	0	Bilateral strip	36.1
11	62.6	Male	22	0	Bilateral strip, right sided resection	51.2
12	62.2	Male	16	0	Bilateral strip	82.8

There was no association between extent of diaphragm intervention and pleuropulmonary recurrence (p>0.05).

Post-operative complications are summarised in Table 1b below. Half of the patients had a respiratory complication, in the form of a pleural effusion, pulmonary embolus or pneumonia. Other complications of note include three patients requiring a return to theatre.

**Table 1b:** Post-operative complications in high grade appendiceal neoplasm patients with chest recurrence.

Patient	Post-operative complications
1	Pulmonary embolus Pain
2	Sepsis Pulmonary effusion Ileus
3	Surgical site infection Dehiscence
4	Intra-operative collection secondary to pancreas leak Post-operative bleed requiring return to theatre Urinary retention and hydronephrosis
5	Liver laceration Pleural effusion Pneumothorax Intra-abdominal collection Sepsis Hospital acquired pneumonia Femoral nerve palsy
6	Pleural effusion Ileus
7	Pulmonary embolus Small bowel obstruction Intra-abdominal collection Intra-abdominal haematoma
8	Pneumonia Intra-abdominal collection Coagulopathy
9	Intra-abdominal infection Intra-abdominal collection Wound dehiscence
10	Ileus Intra-abdominal collection Adrenal haemorrhage
11	Intra-abdominal bleeding, requiring return to theatre Rapid atrial fibrillation Intra-abdominal collection growing multi-resistant staphylococcus aureus (MRSA)
12	Compromised stoma requiring return to theatre Intra-abdominal sepsis and collections requiring aspiration twice

### Mesothelioma

**Diagram 2:**
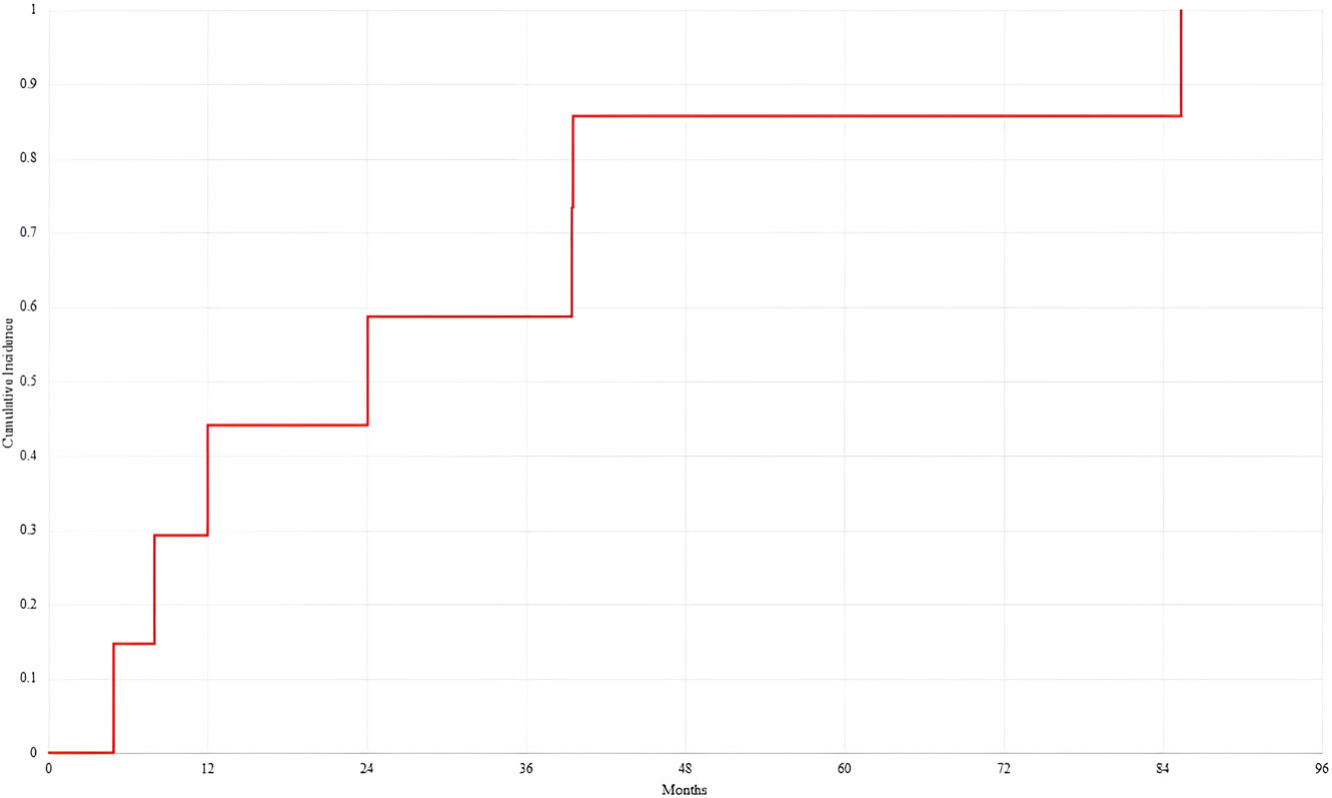
Kaplan–Meier curve demonstrating time till chest disease recurrence for patients with mesothelioma.

Eight patient’s tumour histopathological subtype was mesothelioma (Table 2a). Age of the patients at time of operation ranged from 32.5 to 64.2, with a mean age of 44.9 years. Five (62.5 %) of the patients were male. PCI for the patients ranged from 15 to 30, with a median PCI of 22.1. CC score for all patients were either 0 or 1. Diaphragmatic intervention in one patient was unilateral. All seven other patients had bilateral intervention. Two of the eight patients had resections of the diaphragm, with the rest having stripping only. Time till chest recurrence, in months, ranged from 8.02 to 85.4 months (Diagram 2).

Pre-operative and operative features that were statistically significant include ICU length of stay (r^2^=−0.24, p<0.05), and CC score (r^2^=0.28, p<0.05). There was no significance in regard to operative hours, total hospital length, PCI, gender or age.

There was no association between extent of diaphragm intervention and pleuropulmonary recurrence (p>0.05).

Post-operative complications are summarised in Table 2b. Half (n=4) of the patients had intra-abdominal collections. One patient required a return to theatre and had multiple post-operative complications. Half of the patients also had respiratory complications, in the form of pleural effusion, acute respiratory distress syndrome (ARDS), and pneumothorax.

**Table 2a:** Operative characteristics of mesothelioma patients who had recurrence in the form of chest disease.

Patient	Age (years)	Gender	PCI	CC score	Diaphragmatic intervention	Time till chest recurrence (months)
1	32.5	Female	28	0	Bilateral strip, right sided resection	8.02
2	46.9	Male	20	1	Bilateral strip	38.8
3	51.4	Male	15	0	Bilateral strip	39.46
4	39.1	Female	30	1	Right sided strip	24.05
5	64.2	Male	32	1	Bilateral strip	85.4
6	41.4	Female	16	0	Bilateral strip	39.5
7	48.5	Male	18	1	Left sided strip, right sided resection	4.93
8	35.1	Male	18	0	Bilateral strip	12

**Table 2b:** Post-operative complications in mesothelioma patients with chest recurrence.

Patient	Post-operative complications
1	Pleural effusion
2	Intra-abdominal collection secondary to pancreatic leak Groin collection
3	Intra-abdominal collection secondary to intra-abdominal infection Pneumonia
4	Sepsis Small bowel perforation requiring return to theatre Deep vein thrombosis Intra-abdominal collection Intra-abdominal bleeding Enterocutaneous fistula Pleural effusion
5	Intra-abdominal collection
6	Acute respiratory distress syndrome (ARDS)
7	Pneumothorax Pleural effusion Ileus
8	Pneumothorax Pleural effusion

## Discussion

This investigation demonstrates the various characteristics and post-operative complications associated with patients who underwent diaphragmatic interventions at time of peritonectomy and time till pleural or chest disease recurrence, investigating mesothelioma and high grade appendiceal neoplasms. Nearly all of the patients had some form of respiratory compromise in the post-operative setting. This study did not note any pattern or associated pre-operative features that were associated with pleuropulmonary recurrence. It must be noted that this cohort of patients were taken from a total of 716 and thus is a rare yet important phenomena to be aware of.

Sugarbaker et al. in 2009 [14] underwent an investigation to note the recurrence of pleuropulmonary disease in patients who underwent CRS and hyperthermic intraoperative thoracoabdominal chemotherapy. In this investigation, 30 patients were noted to require diaphragm excision of varying histopathological subtypes. There were three patients who had recurrence within the thorax or pleural.

Median time till chest disease for patients with high grade appendiceal neoplasm was 16.9 months. Another investigation by Kawaguchi et al. [15] for patients with appendiceal neoplasm had median overall survival of 45.5 months. However, mean time till disease recurrence in the chest was not independently noted.

Appendiceal neoplasms and the rates of pleuropulmonary recurrence have been investigated greater than any other form of peritoneal malignancy. An investigation by Beane et al. in 2019 [16] on 382 patients with appendiceal PMP was performed. In this investigation, it was noted that patients who had recurrence in the form of pleuropulmonary disease were associated with diaphragm stripping or resection at the time of surgery, incomplete cytoreduction, high tumour grade, poor performance status, and major morbidity. This is reflected in our investigation, in which all patients had high grade appendiceal neoplasm, underwent diaphragm intervention, and majority had some form of post-operative complication. Unlike the findings by Beane et all, cytoreduction was noted as a CC0-1 for all patients in the high grade appendiceal neoplasms. It has been noted that if there is evidence of visible malignancy in the pleural cavity, intrapleural CRS and simultaneous thoracoabdominal chemoperfusion can reduce the risk of pleuropulmonary recurrence [17].

It should also be noted that in the appendiceal neoplasm cohort, a single patient had early recurrence of approximately 2 weeks. It should be noted that this is likely a patient that had pre-existing microscopic thoracic involvement at time of initial peritonectomy that had not been previously detected.

Of a cohort of 63 patients, 8 (12.7 %) of patients had pleuropulmonary recurrence. Median time till chest disease for patients with mesothelioma was 31.4 months. The pathophysiology of mesothelioma, like other solid tumours, involves the stepwise progression of mitogenic signalling, supress apoptosis, enhance cell growth and replication, genetic instability, tissue invasion and metastasis [18]. Common symptoms of mesothelioma include recurrent pleural effusion. Typically, peritoneal mesothelioma remains confined to the peritoneal cavity [19]. In the literature, seeding post-operatively or radiological intervention ranges from 19–51 % [20]. This suggests that diaphragm intervention does not increase risk of seeding, with appropriate management.

Limitations of our investigation include only reviewing patients with pleuropulmonary disease recurrence in two different histopathological subtypes. Further investigation should be made into comparison with cohorts who did not have pleuropulmonary recurrence. Moreover, this investigation had low number of patients, with subgroup comparison noting statistical underpowering, and thus greater quality would be noted in a study looking at a greater cohort of patients. This study included two vastly different histopathological entities, peritoneal mesothelioma and high-grade appendiceal neoplasms. These two histopathologies are completely separate disease entities, with differing pathophysiology, methods of dissemination, potential treatment options and their response to therapy. By combining these two histopathologies, there are significant limitations due to selection bias and confounding. Due to this, the ability to apply the findings may be limited due to generalisability. Future large scale cohort studies should be performed with cohorts separated by histological disease to validate the observations of this study. Finally, this is a retrospective review and thus the limitations associated with this form of study should be considered.

## Conclusions

This investigation is an early consideration of patients and their demographic features and post-operative complications in patients who had diaphragm stripping and pleuropulmonary recurrence. Due to the low rates of recurrence in this cohort study, limited conclusions regarding recurrence rate can be clearly delineated. Further investigations should be conducted to note the rates of pleuropulmonary recurrence in greater cohorts of patients and the impact of diaphragm intervention on the rate of recurrence.
